# Impacts of the COVID-19 public health emergency on healthcare professional delivery of opportunistic behaviour change interventions: a retrospective cohort study

**DOI:** 10.1186/s12913-023-10522-7

**Published:** 2024-02-05

**Authors:** Chris Keyworth, Mark Conner, Judith Johnson, Tracy Epton, Katharina S. Vogt, Christopher J. Armitage

**Affiliations:** 1 School of Psychology, University of Leeds, LS2 9JT Leeds, UK; 2 Manchester Centre for Health Psychology, School of Health Sciences, University of Manchester, M13 9PL Manchester, UK; 3 Manchester University NHS Foundation Trust, Manchester Academic Health Science Centre, M13 9PL Manchester, UK; 4 NIHR Greater Manchester Patient Safety Research Collaboration, University of Manchester, M13 9PL Manchester, UK; 5 Bradford Teaching Hospitals NHS Foundation Trust, Bradford, UK

**Keywords:** Healthcare professionals, Behaviour change interventions, COVID-19, COM-B

## Abstract

**Background:**

The public health policy “Making Every Contact Count” (MECC) compels healthcare professionals to deliver health behaviour change interventions during routine consultations. As healthcare systems continue their recovery from the impacts of the COVID-19 public health emergency, supporting people to modify health behaviours is more important now than when the policy was introduced. The present study aims to: (a) examine changes in healthcare professionals’ awareness of, and engagement with the policy over a five-year period, (b) examine the psychosocial drivers associated with delivering behaviour change interventions, and (c) identify targets to increase healthcare professionals’ delivery of interventions.

**Methods:**

Comparison of data from two independent representative surveys of NHS healthcare professionals working in the UK. In both surveys (July-September 2017; *N* = 1387, and February-March 2022; *N* = 1008), participants were asked to report: (1) awareness of the MECC policy, (2) the prevalence of MECC-related practice (perceived patient benefit, how often interventions were delivered, and time spent delivering interventions), and (3) perceptions of capabilities, opportunities and motivations to deliver behaviour change interventions. T- tests (independent-samples), MANOVA, multiple linear regression, and chi-square analyses were used to generate comparisons between the surveys.

**Results:**

Awareness of the policy increased from 2017 (31.4%) to 2022 (52.0%). However, in 2022 compared with 2017, healthcare professionals reported (a) fewer patients would benefit from behaviour change interventions (49.1% versus 55.9%), (b) they delivered behaviour change interventions to a lower proportion of patients (38.0% versus 50.0%), and (c) they spent a lower proportion of the consultation time delivering interventions (26.5% versus 35.3%). Further, in 2022, compared with 2017, healthcare professionals reported fewer physical opportunities, fewer social opportunities, and fewer psychological capabilities to deliver behaviour change interventions. In the 2022 survey, perceptions of patient benefit and delivery of interventions was associated with greater perceptions of opportunities and motivations.

**Conclusions:**

Health behaviour change interventions remain an important part of routine healthcare in the continued recovery from COVID-19 public health emergency, however reported engagement with MECC-related practices appears to have reduced over time. Future research should consider how healthcare professionals identify patients who might benefit from opportunistic behaviour change interventions, and to increase capabilities, opportunities, and motivations to deliver interventions during routine consultations.

## Background

Healthcare professionals are well placed to support health behaviour change with patients due to their frequent one-to-one patient contact, and they are a trusted and expected source of behaviour change advice [1–4]. There is evidence that healthcare professionals value behaviour change interventions as an important clinical activity [5, 6], and patients welcome interventions during routine consultations [4].

Public health policies are used to compel healthcare professionals to deliver health behaviour change interventions during routine practice [7, 8, 9]. Making Every Contact Count (MECC), a National Health Service (NHS) policy aimed at patient-facing healthcare professionals, places prevention at the centre of every NHS patient -healthcare professional contact [10, 11]. The policy, which is based on behaviour change evidence [12] and developed with partner organisations including local authorities and the National Institute for Health and Care Excellence (NICE) [11], compels healthcare professionals to offer health behaviour change interventions (e.g., smoking cessation, improving diet, increasing physical activity, and reducing alcohol intake) to encourage patients to change their behaviour and to direct them to local services that can support them. Whilst NHS organisations are contractually obliged to implement the policy, previous research shows: (a) lack of awareness of the policy across all healthcare professional groups, and (b) low levels of practice consistent with the policy [5]. A national survey administered in 2017 showed that 31% of healthcare professionals reported having heard of the MECC policy. In the survey, healthcare professionals believed that 56% of the patients they saw in a typical week would benefit from a behaviour change intervention, however they felt unable to “Make Every Contact Count” in 50.0% of these cases [5].

The impacts of the COVID-19 public health emergency on healthcare professionals and organisations have been significant. Internationally, this has included understaffing [13], a perceived fear of becoming infected with the virus [14], and dealing with a lack of personal protective equipment [15]. In the UK, research suggests healthcare professionals have faced a number of challenges during the pandemic, including inadequate training, a lack of consistent guidelines with respect to caring for patients during the pandemic, a changing and challenging work environment [16, 17], and a rapid shift to remote consultations [18, 19]. Consequently, this may have led to changes in the way behaviour change interventions are delivered, as they may have become less of a priority during routine consultations.

These added pressures may have led the MECC policy to be overlooked [20]. The COVID-19 public health emergency has increased health inequalities [21], and may have led to increased alcohol intake, reductions in physical activity, and lower diet quality, compared to pre-pandemic levels [22, 23]. Consequently, supporting people to modify health behaviours is more important now than when the policy was introduced. There is therefore an urgent need to understand how awareness and prevalence of policy-related practice may have been impacted by the pandemic.

As well as examining perceptions of patient benefit of behaviour change interventions and perceptions of reported practice with respect to delivering behaviour change interventions, it would also be valuable to examine whether specific psychosocial drivers are associated with delivering interventions. Michie et al’s [24] Capabilities, Opportunities and Motivations model of Behaviour change (COM-B), which is endorsed by the UK National Institute for Health and Care Excellence [25], is designed to capture the key drivers of behaviour. Whilst there is evidence from previous studies that these proposed drivers of behaviour– perceived personal capability, access to opportunities and personal motivation– do indeed influence the extent to which healthcare professionals deliver behaviour change interventions [26], no research to-date has examined whether these drivers are still important factors in a post-COVID-19 NHS context. This is important because this may allow targeted interventions to be delivered to enhance psychosocial drivers of healthcare professional behaviour.

The present study aims to: (a) examine changes in healthcare professionals’ awareness of, and engagement with the policy over a five-year period (2017–2022), (b) examine the psychosocial drivers associated with delivering behaviour change interventions, and (c) identify targets for interventions to increase healthcare professionals’ delivery of interventions.

## Methods

### Design

We conducted a retrospective cohort study using two cross-sectional online surveys administered by YouGov, a survey panel company, to two independent, representative samples of the UK’s National Health Service (NHS) healthcare professionals pre COVID-19 (in 2017) and during COVID-19 (in 2022).

### Participants

The first survey was conducted in the UK in July-September 2017 with a sample of 1387 healthcare professionals working in the NHS [5]. The second survey (during COVID-19) was conducted in the UK in February-March 2022 (a time which proceeded a COVID-19 ‘peak’ when one in 23 people in the UK had the virus (up from 1 in 70 in December 2021) [27]) on a sample of 1008 healthcare professionals working in the NHS.

In both surveys a range of patient-facing healthcare professionals were recruited and included general practitioners (GPs); specialist doctors; nurses; midwives, and scientific, therapeutic and technical staff (e.g. pharmacists, psychologists, speech and language therapists). The sampling frame aimed to obtain the widest possible variation in participants according to demographic characteristics.

### Procedure

Participants were identified from a pre-existing sample of healthcare professionals recruited and incentivised by YouGov to complete the questionnaire (respondents accumulate points for taking part in surveys, which can be exchanged for cash or entry into a prize draw). The first survey was part of a wider study examining the barriers and enablers to healthcare professionals delivering behaviour change interventions [5], and the second survey was part of a wider questionnaire aimed at developing and piloting a theory-based intervention for healthcare professionals. Ethical approval was obtained from the University of Manchester Research Ethics Committee (2017 survey ref: 2017-0739-1780) and the University of Leeds Research Ethics Committee (2022 survey ref: PSYC-398) and participants gave informed consent at the beginning of the surveys.

### Measures

Both questionnaires collected demographic information such as gender and age, healthcare setting (e.g. primary care, secondary care) as well as the number of patients seen by the healthcare professional in a typical week. Participants were asked about their awareness of the MECC policy (using a yes/no response option; a description of which was provided after participants answered, in order not to influence the participant’s response) and about the extent to which they engaged in this activity as part of their daily practice. Participants were asked to rate (using a 0–100% rating scale): (a) what proportion of patients they saw would benefit from opportunistic behaviour change interventions, (b) the proportion of times they delivered opportunistic behaviour change interventions to the patients they thought would benefit, and (c) how much of their contact time they spent delivering opportunistic behaviour change interventions to the patients they thought would benefit. Keyworth et al.’s brief COM-B measure [26] was used to assess healthcare professionals’ capabilities, opportunities and motivations, which comprises six items designed to measure physical capability, psychological capability, physical opportunity, social opportunity, reflective motivation, and automatic motivation (presented in full in Table 1). Each item is accompanied with a brief definition of each construct (e.g., the physical opportunity item is accompanied with: What is physical opportunity? The environment provides the opportunity to engage in the activity concerned (e.g., sufficient time, the necessary materials, reminders). The physical opportunity and social opportunity items are measured using a 0–100% rating scale, and the physical capability, psychological capability, reflective motivation, and automatic motivation items are assessed on 11-point scales (strongly disagree[0]-strongly agree[10]).

**Table 1:** Comparison of capabilities, opportunities, and motivations between 2017 and 2022 survey respondents

	Pre-COVID-19 survey (2017)		During COVID-19 survey (2022)		Univariate ANOVA				
Question	Mean (%)	(SD)	Mean (%)	(SD)	df	Df E	F	p	η_p_^2^
Physical capability: “I am PHYSICALLY able to Make Every Contact Count”^b^	6.69	3.10	6.68	2.65	6	1747	0.005	0.94	0.000
Psychological capability: “I am PSYCHOLOGICALLY able to Make Every Contact Count What is psychological capability?”^b^	7.21	2.64	6.69	2.53	6	1747	17.185	< 0.001	0.010
Physical opportunity: “Of the service users you see in a typical working week, with what proportion do you have the PHYSICAL opportunity to Make Every Contact Count?”^a^	46.39	33.19	37.41	35.98	6	1747	28.461	< 0.001	0.016
Social opportunity: “Of the service users you see in a typical working week, with what proportion do you have the SOCIAL opportunity to Make Every Contact Count?”^a^	43.58	32.25	33.32	33.47	6	1747	41.55	< 0.001	0.023
Reflective motivation: “I am motivated to Make Every Contact Count”^b^	6.52	2.83	6.43	2.72	6	1747	0.511	0.48	0.000
Automatic motivation: “Making Every Contact Count is something I do automatically”^b^	6.38	3.01	6.24	2.83	6	1747	0.927	0.34	0.001

^a^using a 0–100% rating scale; ^b^assessed on 11-point scales (strongly disagree[0]-strongly agree[10]

### Analyses

Descriptive statistics were used to quantify MECC-related activities, and mean ratings of awareness of the MECC policy and MECC-related practices (the proportion of patients that would benefit from behaviour change interventions, the proportion to whom they deliver interventions and the amount of time spent on this activity) were calculated. *T*- tests (independent-samples), MANOVA and chi-square was used for comparisons between the surveys. Chi-square was used to gauge the representativeness of survey two (conducted in 2022) compared to survey one (conducted in 2017). MANOVA was used to compare mean scores for capabilities, opportunities, and motivations between 2017 survey respondents and 2022 survey respondents. A series of multiple linear regressions were used to examine associations between sociodemographic factors, psychosocial variables, and delivery of behaviour change interventions (among the 2022 survey respondents only). Separate linear regression models were conducted for each of the three dependent variables: perceptions of patient benefit of interventions, delivery of interventions, and time spent delivering interventions. Each model was adjusted for potential correlates of delivering interventions (age, gender, and ethnicity).

## Results

### Sample characteristics

Table 2 shows an overview of data from survey two (2022) compared to survey one (2017). The two samples did not differ with respect to gender (*p*’s > 0.05), age (*p* =.91), setting (*p*’s > 0.05), and most healthcare professional groups (*p*’s > 0.05). We were unable to recruit comparable numbers of GPs; consequently, we obtained an over-representation of staff in the category “Other Hospital and Community Health Services (HCHS) staff/unknown classifications”. Table 2 also shows the reported number of: (a) service users seen in a typical week, and (b) the mean time spent with each patient. Independent samples *t*-tests indicated that in a typical week, healthcare professionals in the 2022 survey (*M* = 33.39, *SD* = 30.23), compared with healthcare professionals in the 2017 survey (*M* = 50.00, *SD* = 31.89) reported seeing fewer patients (*p* <.001). There were no differences between the 2022 survey respondents (*M* = 32.00, *SD* = 20.40), and 2017 survey respondents (*M* = 31.00, *SD* = 19.54) in the reported number of minutes spent with each patient (*p* =.06).

**Table 2:** Sample characteristics (2017 sample compared to 2022 sample)

		Pre-COVID-19 survey (2017)			During COVID-19 survey (2022)					
Variable	*n*	(%)	Mean	(SD)	*n*	(%)	Mean	(SD)	X2 for difference between two samples	*t*-test
Gender (%)										
Male Female Total	446 941 1387	(32.2) (67.8)			266 741 1008	(26.4) (73.5)			0.87 (*p* =.35) 0.87 (*p* =.35)	
Age, years			45	(11.46)			45	(11.97)		0.11
Healthcare professional group^a^										
General Practitioners Specialist doctors Nurses and health visitors Midwives Ambulance staff Scientific, therapeutic and technical staff^b^ Nurses working in GP practices Support to clinical staff Other HCHS staff/unknown classifications Total	332 125 438 42 20 270 88 49 23 1387	(23.9) (9.0) (31.6) (3.0) (1.4) (19.5) (6.3) (3.5) (1.3)			45 146 409 30 10 143 46 58 121 1008	(4.5) (14.5) (40.6) (3.0) (1.0) (14.2) (4.6) (5.8) (12.0)			14.56 (*p* <.01) 1.70 (*p* =.19) 1.75 (*p* =.19) 1.00 (*p* = 1.00) 1.00 (*p* = 1.00) 1.28 (*p* =.26) 0.10 (*p* =.76) 0.42 (*p* =.52) 9.95 (*p* <.01)	
Setting										
NHS Acute Care NHS Tertiary Care NHS Community Care NHS Primary Care Other Total	576 83 257 339 132 1387	(41.5) (6.0) (18.5) (24.4) (9.5)			414 89 230 193 82	(41.1) (8.8) (22.8) (19.1) (8.2)		-	0.02 (*p* =.89) 0.65 (*p* =.42) 0.48 (*p* =.49) 0.23 (*p* =.63) 0.24 (*p* =.62)	
How many service users do you see in a typical week?			50	(31.89)			33.39	(30.23)		12.48***
Total number of service users seen by all included healthcare professionals			58,906^b^		33,631^b^					
How many minutes do you spend on average with each service user? (mins)			31	(19.54)			32	(20.40)		-1.92
Before today, had you heard of the Making Every Contact Count consensus statement?										
Yes	436	(31.4)			524	(52.0)			9.08 (*p* <.01)	
No	830	(59.8)			484	(48.0)			2.90 (*p* =.09)	
Do not know	83	(6.0)			-	-				
Did not state	38	(2.7)			-	-				
Total	1387				1008					

***p <.001; **p <.01; *p <.05

^a^ Staff categories and NHS data according to NHS digital workforce statistics (headcount), excludes NHS infrastructure support and admin staff; https://digital.nhs.uk/data-and-information/publications/statistical/healthcare-workforce-statistics/healthcare-workforce-statistics-march-2017-experimental

^b^ Participants were asked to estimate the number of patients they would see in a typical week; therefore, this is an approximate number only, based on n = 1177 healthcare professionals who provided an estimate

### Awareness and prevalence of “making every contact count” (2022 survey versus 2017 survey)

**Fig. 1:**
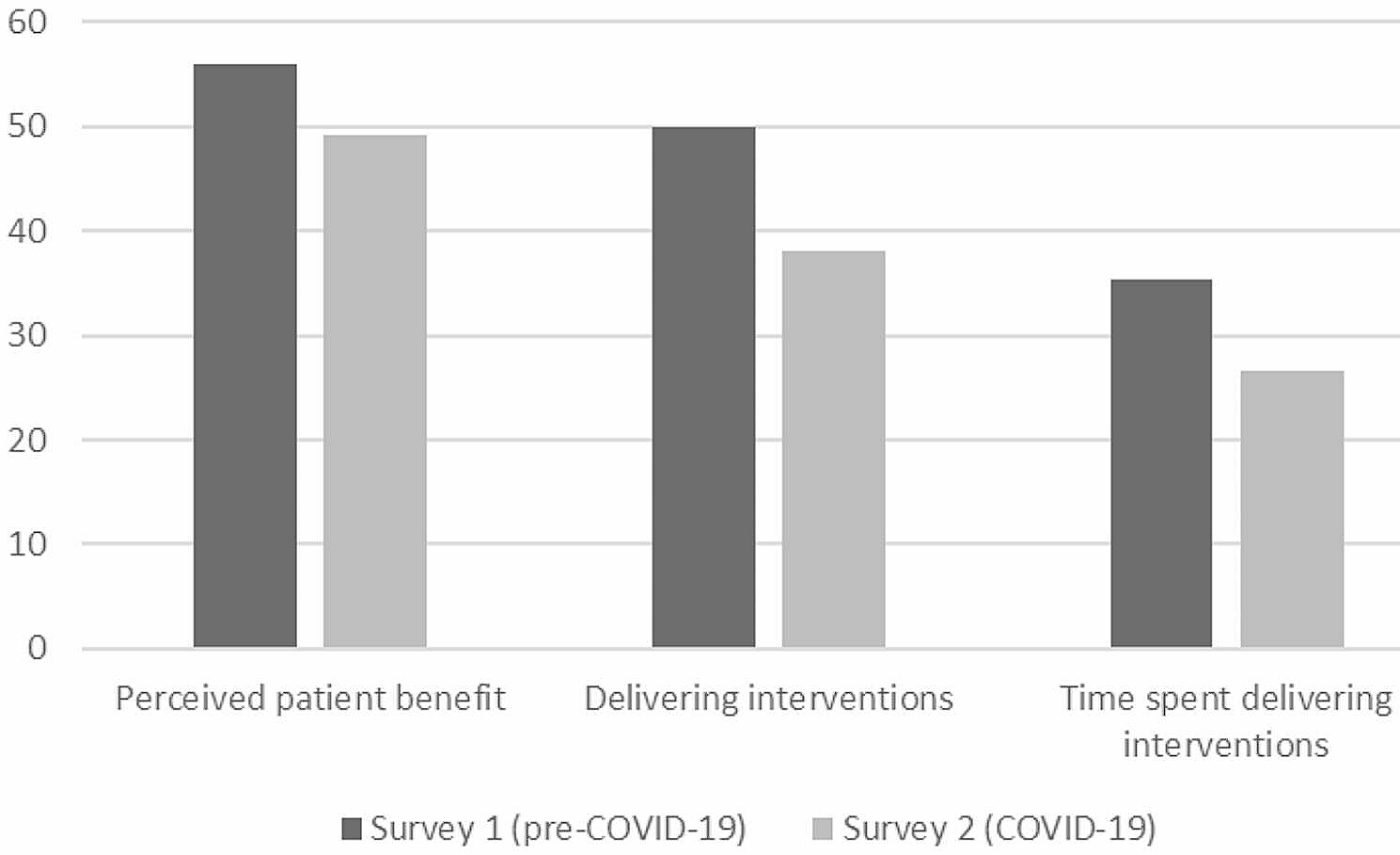
Comparisons of proportion and awareness of Making Every Contact Count

A higher proportion of healthcare professionals had heard of the “Making Every Contact Count” policy in the 2022 survey (52%), compared with the 2017 survey (31.4%) (*X*^2^ values are presented in Table 2). Using Pillai’s trace, MANOVA indicated that there were significant differences between the 2022 survey respondents and the 2017 survey respondents in terms of prevalence of Making Every Contact Count-related practices (V = 0.031, *F*(3, 1892) = 20.45, *p* <.001, η_p_^2^ = 0.03). Univariate *F* tests revealed significant reductions between groups (in 2022 compared with 2017) for: (1) the perceived proportion of patients who would benefit from behaviour change interventions (2022 survey *M* = 49.11, *SD* = 35.64, versus 2017 survey *M* = 55.93, *SD* = 31.86; *p* <.001, (2) the proportion of patients to whom healthcare professionals delivered interventions (2022 survey *M* = 38.00, *SD* = 36.33, versus 2017 survey *M* = 50.00, *SD* = 31.34; *p* <.001, and (3) the proportion of the consultation time spent delivering behaviour change interventions (2022 survey *M* = 26.54, *SD* = 32.68, versus 2017 survey *M* = 35.30, *SD* = 30.92; *p* <.001 (see Table 3). The results indicate that the implementation of “Making Every Contact Count” decreased in 2022, compared with 2017. Therefore, there is scope to improve the prevalence of behaviour intervention delivery amongst healthcare professionals. A comparison of the prevalence of “Making Every Contact Count” across the two surveys is presented in Fig. 1.

**Table 3:** Difference between groups for awareness and prevalence of “Making Every Contact Count” (2017 survey; *n* = 1387, versus 2022 survey; *n* = 1008)

	Pre-COVID-19 survey (2017)		During COVID-19 survey (2022)		Univariate ANOVA				
Question	Mean (%)	(SD)	Mean (%)	(SD)	df	Df E	F	p	η_p_^2^
Of the service users you see in a typical working week, what proportion **do you think would benefit** from you Making Every Contact Count?	55.93	(31.86)	49.11	(35.64)	1	1894	14.771	< 0.001	0.008
Of the service users you see in a typical working week, who you think would benefit, **with what proportion do you** Make Every Contact Count?	50.00	(31.34)	38.00	(36.33)	1	1894	54.332	< 0.001	0.028
Of the service users you see in a typical working week who you think would benefit, **how much of their appointment time** do you spend with them making every contact count?	35.30	(30.92)	26.54	(32.68)	1	1894	36.446	< 0.001	0.019

***p <.001

### Perceived capabilities, opportunities, and motivations to deliver behaviour change interventions

Using Pillai’s trace, MANOVA indicated that there were significant differences between the 2022 survey respondents and the 2017 survey respondents in perceived capabilities, opportunities, and motivations to deliver behaviour change interventions (V = 0.045, *F*(6, 1747) = 13.87, *p* <.001, η_p_^2^ = 0.05). Univariate *F* tests revealed significant differences between groups for: psychological capability (2022 survey *M* = 6.69, *SD* = 2.53, versus 2017 survey *M* = 7.21, *SD* = 2.64; *p* <.001, physical opportunity (2022 survey *M* = 37.41, *SD* = 35.98, versus 2017 survey *M* = 46.39, *SD* = 33.19; *p* <.001, and social opportunity (2022 survey *M* = 33.32, *SD* = 33.47, versus 2017 survey *M* = 43.58, *SD* = 32.25; *p* <.001 (see Table 1). The results indicate that healthcare professionals reported fewer physical opportunities, fewer social opportunities, and fewer psychological capabilities to deliver behaviour change interventions in 2022, compared with 2017. There were no significant differences between groups for: physical capability (2022 survey *M* = 6.68, *SD* = 2.65, versus 2017 survey *M* = 6.69, *SD* = 3.10; *p* =.94, reflective motivation (2022 survey *M* = 6.43, *SD* = 2.72, versus 2017 survey *M* = 6.52, *SD* = 2.83; *p* =.48, and automatic motivation (2022 survey *M* = 6.24, *SD* = 2.83, versus 2017 survey *M* = 6.38, *SD* = 3.01; *p* =.34. Therefore, there is scope to improve opportunities and capabilities amongst healthcare professionals. A comparison of the perceived capabilities, opportunities, and motivations across the two surveys is presented in Table 1.

### Associations between sociodemographic variables, capabilities, opportunities, and motivations to deliver behaviour change interventions (2022 survey respondents)

Multiple linear regression (Table 4) showed that, controlling for sociodemographic variables, healthcare professionals’ perceptions of their opportunities and motivations were significantly associated with all three dependent variables. More specifically, higher proportions of perceived patient benefit, higher prevalence of delivering behaviour change interventions, and greater amount of reported time delivering interventions were each associated with higher levels of physical and social opportunity, and higher levels of reflective and automatic motivation to deliver interventions.

**Table 4:** Associations between sociodemographic variables, COM-B and delivering behaviour change interventions (*2022 survey respondents only*)

Variable	*B*	*SE*	*95% CI*	*p*
*Of the service users you see in a typical working week, what proportion do you think would benefit from you Making Every Contact Count?*				
Gender (1 = Men; 2 = Women)	-0.00	2.19	-4.41, 4.19	0.96
Age	0.05	0.08	-0.02, 0.30	0.08
Ethnicity (1 = White; 2 = Black, Asian or Minority Ethnic)	-0.03	3.02	-8.88, 2.98	0.33
Physical Capability	-0.08	0.59	-2.27, 0.02	0.05
Psychological Capability	-0.02	0.62	-1.47, 0.98	0.69
Physical Opportunity	0.18	0.04	0.10, 0.26	< 0.001
Social Opportunity	0.17	0.04	0.10, 0.26	< 0.001
Reflective Motivation	0.27	0.59	2.42, 4.73	< 0.001
Automatic Motivation	0.11	0.55	0.32, 2.46	< 0.05
*Of the service users you see in a typical working week, who you think would benefit, with what proportion do you Make Every Contact Count?*				
Gender (1 = Men; 2 = Women)	0.05	2.10	-0.44, 7.81	0.08
Age	0.04	0.08	-0.02, 0.28	0.09
Ethnicity (1 = White; 2 = Black, Asian or Minority Ethnic)	-0.01	2.90	-6.48, 4.89	0.78
Physical Capability	-0.06	0.04	-1.91, 0.29	0.15
Psychological Capability	0.00	0.04	-1.14, 1.21	0.95
Physical Opportunity	0.17	0.57	0.09, 0.24	< 0.001
Social Opportunity	0.25	0.52	0.19, 0.35	< 0.001
Reflective Motivation	0.18	0.56	1.22, 3.44	< 0.001
Automatic Motivation	0.20	0.60	1.52, 3.58	< 0.001
Of the service users you see in a typical working week who you think would benefit, how much of their appointment time do you spend with them making every contact count?				
Gender (1 = Men; 2 = Women)	0.04	1.96	-0.61, 7.08	0.10
Age	0.04	0.07	-0.05, 0.24	0.19
Ethnicity (1 = White; 2 = Black, Asian or Minority Ethnic)	0.02	2.71	-3.75, 6.86	0.57
Physical Capability	-0.08	0.52	-2.04, 0.01	0.05
Psychological Capability	0.01	0.56	-1.00, 1.18	0.87
Physical Opportunity	0.18	0.04	0.09, 0.53	< 0.001
Social Opportunity	0.25	0.04	0.17, 0.32	< 0.001
Reflective Motivation	0.14	0.53	0.63, 2.70	< 0.01
Automatic Motivation	0.19	0.49	1.17, 3.09	< 0.001

## Discussion

### Principal findings

Across the two surveys, awareness of the public health policy Making Every Contact has increased among healthcare professionals in 2022 (52.0%) compared with 2017 (31.4%). Despite this, reported engagement with policy-related practices appears to have reduced over time. Across the two surveys, healthcare professionals: (a) reported fewer patients would benefit from behaviour change interventions, (b) reported delivering behaviour change interventions to a lower proportion of patients, and (c) spent a lower proportion of the consultation time delivering behaviour change interventions. Perceptions of opportunities and motivations were associated with delivery of behaviour change interventions.

### Comparison with existing literature

Previous work suggests that healthcare professionals are willing and see the value of delivering behaviour change interventions as part of routine clinical interactions with patients, in accordance with public health strategies [5, 6, 11]. Despite the increasing levels of awareness of policy observed in the present study, the falling levels of policy-related practices is a cause for concern for both policy makers and intervention developers. Findings from the present study suggest that healthcare professionals perceived fewer patients would benefit from behaviour change interventions in 2022 compared with 2017. This is in contrast with recent literature suggesting that for some people, the pandemic may have led to increased alcohol intake, reductions in physical activity, and lower diet quality, compared to pre-pandemic levels [22, 23]. Consequently, supporting people to modify health behaviours should continue to be an important area of healthcare delivery.

A concerning observation are the findings with respect to falling levels of policy-related practice amongst healthcare professionals who are well placed to support behaviour change with patients, due to their frequent one-to-one contact with patients. For example, settings such as primary care [28] and maternity services [29] offer teachable moments to address behaviour change, yet our findings suggest significant reductions in delivery of behaviour change interventions by GPs, nurses and health visitors, nurses working in GPs practices, and midwives across the two surveys. Similar findings were observed for time spent delivering interventions, with nurses and health visitors, midwives, ambulance staff, scientific, therapeutic and technical staff, nurses working in GP practices, and staff providing support to clinical staff all reporting spending lower proportions of the consultation time delivering behaviour change interventions. The present study also provides additional support for the predictive validity of the COM-B model, which has shown to predict several behaviours across a diverse range of contexts [26, 30, 31].

Specific reasons contributing to the likelihood of behaviour change being part of routine medical consultations may be due to the added pressures of the pandemic, which may have resulted in practice consistent with the Making Every Contact Count policy being over-looked. Specific challenges may have included dealing with the shift to remote consultations [32], the influx of COVID-19 patients, and understaffing which may have been caused due to staff illness [33].

It may also be the case that fewer patients are being seen by healthcare professionals since the onset of the pandemic. For example, data from the earlier stages of the pandemic showed a reduction in consultation rates in the four months following the onset of the pandemic, compared with the previous year, as a result of the shift to remote consultations [18]. Some patients may also have been discouraged from consulting with the healthcare service in response to public health messages encouraging people to ease the burden on health services [34]. This finding is consistent with our data: GPs, specialist doctors, nurses and health visitors, and ambulance staff were among the groups who reported seeing fewer patients in 2022, compared with 2017.

### Implications for research and practice

It would be valuable to explore specific drivers of healthcare professionals’ capabilities, opportunities, and motivations to deliver behaviour change interventions during routine medical consultations, in order to understand the policy and practice recommendations required to promote greater uptake of policy-related practices [24]. In the present study, we observed lower levels of physical opportunities and social opportunities following the onset of the pandemic, compared to pre-pandemic levels. This may suggest two areas for future research. First, our findings suggest that helping facilitate an environment conducive to having discussions about behaviour change and providing the specific means of delivering brief behaviour change interventions in a time-restricted consultation (physical opportunities) may encourage more healthcare professionals to deliver interventions. Second, encouraging organisations to provide support for healthcare professionals to deliver interventions, and thereby creating social and cultural norms (social opportunity) may be an important focus of future work. As one clear post-pandemic change in healthcare delivery is an increase in remote consultations, it may be valuable for future studies to focus on this and explore how remote consultations may impact on the delivery of behaviour change interventions. It could be suggested that the lack of extra cues in the online environment may reduce: (1) healthcare professionals’ awareness of patient need for behaviour change, and (2) opportunities to highlight patient benefit and explore this with patients. This possibility is supported by evidence that dementia diagnosis rates have dropped following increases in remote general practice consultations, but the mechanisms underlying this remain unclear [35].

Future work should aim to develop and test behaviour change interventions that can be incorporated into time-restricted medical consultations and take into account the added complexities of healthcare delivery in light of the changes following the onset of the pandemic [18, 19]. Another possible area for future research would be to help support healthcare professionals to identify patients who would benefit from behaviour change interventions, and to provide the means of engaging patients in discussions about health behaviours (healthcare professionals: [a] perceived fewer patients would benefit from interventions in 2022, compared with 2022, and [b] did not deliver interventions on 62% of occasions in which they perceived a need). Supporting healthcare professionals to identify the barriers to engaging patients in discussions about behaviour change, and potential enablers to increase delivery of interventions, should be a focus of future research.

### Strengths and limitations

To our knowledge, this is the first attempt to examine the awareness and prevalence of “Making Every Contact Count”-related activities across healthcare professional groups using two large national surveys, across two different times. Findings highlight important opportunities to support healthcare professionals to deliver behaviour change interventions considering the ever-changing landscape of healthcare delivery, as a result of the changes in response to the pandemic. We have identified potential targets to drive healthcare professional behaviour change, and whilst our measures were self-reported, prior work shows the tool is reliable and valid, and is sensitive to COVID-19-related changes [26, 36]. Future research should aim to build on these findings and develop and test brief interventions designed to support healthcare professionals in the area of clinical practice.

There are limitations to this study. Although the two samples were large and both intended to be comparable to the patient-facing NHS healthcare professional workforce, we were unable to follow-up the respondents in our original survey [5]. This was primarily due to respondents being members of YouGov’s pre-existing panel, members of which may have changed in the five years since the original survey was conducted. Further, the sample may not be fully representative of the healthcare professionals working in the NHS as a whole. In both surveys however, YouGov attempted to overcome this by seeking the widest possible variation in participants according to demographic characteristics. Additionally the cross-sectional nature of the study meant that we were unable to track any changes over time in our respondents, and causality cannot be inferred. Other COVID-19-related factors that may not have been captured in the present study may be contributing to whether brief behaviour change interventions are delivered to patients. For example, there may be different priorities across the various healthcare specialisms during the pandemic, particularly those professionals working in secondary or specialist care roles, who may have more focused consultations which may have been prioritising treating COVID-19 patients [37] and may perceive a lack of time to deliver opportunistic behaviour change interventions. Whilst this a limitation of cross-sectional survey designs, we are now undertaking qualitative work that aims to capture the most relevant barriers and enablers to healthcare practice during the pandemic, beyond those commonly reported in the literature, such as time, workload and organisation barriers [6].

## Conclusions

Healthcare professionals value and are aware of the importance of delivering behaviour change interventions during routine medical consultations. Despite growing awareness of policy, our findings suggest that the COVID-19 public health emergency may have led to changes in the way behaviour change interventions are delivered. Further research is needed to consider how healthcare professionals identify patients who might benefit from opportunistic behaviour change interventions and focus on the development of behaviour change interventions aimed at increasing healthcare professionals’ opportunities and motivations to deliver behaviour change interventions during routine medical consultations.

## Data Availability

The datasets used and/or analysed during the current study are available from the corresponding author on reasonable request.
